# Effects of Chondroitin Sulfate on Joint Symptoms and Physical Function in Adults with Mild Knee Osteoarthritis: A Randomized, Double-Blind, Placebo-Controlled Trial

**DOI:** 10.3390/nu18203391

**Published:** 2026-10-16

**Authors:** Hyang-Im Baek, Soo Jung Park

**Affiliations:** 1 Department of Food Science & Nutrition, Woosuk University, Wanju 55338, Republic of Korea; hyangim100@gmail.com; 2 Department of Sasang Constitutional Medicine, College of Korean Medicine, Woosuk University, Jeonju 55338, Republic of Korea

**Keywords:** chondroitin sulfate, knee osteoarthritis, joint pain, physical function, joint health, functional food

## Abstract

**Background/Objectives**: Although chondroitin sulfate (CS) is commonly used for knee osteoarthritis (OA), evidence regarding its effects on joint symptoms and physical function remains inconsistent. This study aimed to evaluate the effects of CS supplementation on joint symptoms and physical function in adults with mild knee OA. **Methods**: In this 12-week, randomized, double-blind, placebo-controlled trial, 100 adults with mild knee OA were randomly assigned to receive CS (1200 mg/day; *n* = 50) or placebo (*n* = 50). The primary outcomes were the Korean Western Ontario and McMaster Universities Osteoarthritis Index (K-WOMAC) total and subscale scores. Secondary outcomes comprised the pain visual analog scale (VAS), 20-m walking time, Korean Australian/Canadian Osteoarthritis Hand Index (K-AUSCAN), and grip and pinch strength. **Results**: At week 12, the reduction in the K-WOMAC total score was significantly greater with CS than with placebo (−19.26 ± 2.74 vs. −2.10 ± 1.37; *p* < 0.001), along with greater reductions in the pain, stiffness, and physical function subscale scores (all *p* < 0.001). Notable improvements were also observed in the pain VAS (−18.82 ± 3.50 vs. −3.04 ± 3.10 mm) and 20-m walking time (−1.40 ± 0.62 vs. 0.23 ± 0.32 s) (both *p* < 0.001). K-AUSCAN scores and grip and pinch strength also improved significantly compared to placebo. No clinically meaningful safety concerns were observed. **Conclusions:** These findings suggest that CS supplementation may be beneficial in improving joint symptoms and physical function in adults with mild knee OA, demonstrating consistent benefits across both self-reported outcomes and objective walking performance.

## 1. Introduction

Osteoarthritis (OA) is a common chronic joint disorder characterized by progressive structural and functional changes that contribute to pain, stiffness, and impaired physical function [1,2]. OA commonly affects weight-bearing joints such as the knee, but its clinical burden extends beyond pain alone, since limitations in mobility and in the ability to perform daily activities can substantially affect quality of life [1,2]. OA also imposes a considerable individual and socioeconomic burden, and this burden is expected to increase further with population aging and the rising prevalence of major risk factors such as obesity [1,3,4]. Given the chronic and progressive nature of OA, managing joint symptoms and maintaining physical function may be important even in individuals with relatively mild disease.

Current management of OA involves a combination of non-pharmacological and pharmacological approaches tailored to disease severity, individual symptoms, and comorbidities [5,6]. Exercise, weight management, and patient education are widely recommended as core components of OA management, whereas nonsteroidal anti-inflammatory drugs (NSAIDs) and other pharmacological interventions are commonly used when additional symptom control is required [5,6]. Because OA is a chronic condition requiring long-term management, however, prolonged pharmacological treatment may be limited by potential adverse effects and by individual comorbidities. These considerations have contributed to continued interest in complementary and nutritional approaches to the management of OA symptoms, among which chondroitin sulfate (CS) has received considerable attention [7].

Chondroitin sulfate is a sulfated glycosaminoglycan and a major component of the extracellular matrix of articular cartilage, in which it contributes to the structural and functional properties of the tissue [8,9]. Beyond this structural role, experimental evidence suggests that CS may influence biological processes involved in OA, among them inflammatory responses and the regulation of cartilage matrix metabolism [8,9]. Preclinical evaluation of the present CS preparation showed modulation of matrix metalloproteinases (MMPs) and tissue inhibitor of metalloproteinases 1 (TIMP-1) in a monosodium iodoacetate (MIA)-induced OA model, which suggests a potential role in regulating cartilage matrix degradation [10]. These biological and preclinical findings provide a rationale for further evaluation of the clinical effects of CS on joint symptoms and function.

Several randomized controlled trials have evaluated the clinical effects of CS in individuals with knee OA, but the findings have not been entirely consistent. Zegels et al. reported that 3 months of CS supplementation at 1200 mg/day significantly improved pain and the Lequesne index (LI) relative to placebo in individuals with symptomatic knee OA [11], and Uebelhart et al. evaluated CS in a similar population using the LI and pain VAS together with walking time as a performance-based measure [12]. The large Glucosamine/chondroitin Arthritis Intervention Trial (GAIT), by contrast, found no significant benefit of CS alone at 1200 mg/day over placebo for the primary pain-response outcome in the overall study population [13]. Systematic reviews and meta-analyses have likewise suggested potential benefits of CS for pain and function, while noting substantial heterogeneity across clinical trials [7,14]. Differences in participant characteristics, baseline symptom severity, intervention duration, CS preparation, and outcome measures may account in part for these inconsistent findings [14].

Despite the accumulated clinical evidence, data on the effects of CS specifically in individuals with relatively mild knee OA remain limited, particularly where joint symptoms are considered together with multiple aspects of physical function. Previous trials have relied primarily on pain measures and on self-reported or composite functional indices [11,13], although objective performance-based outcomes such as walking time have been evaluated in some studies [12,15]. Integrating self-reported outcomes with objective measures of mobility and strength may therefore provide a more comprehensive assessment of the functional response to CS. This randomized, double-blind, placebo-controlled trial accordingly aimed to evaluate the effects of 12 weeks of CS supplementation at 1200 mg/day on joint symptoms and physical function in adults with mild knee OA, using complementary self-reported and objective assessments.

## 2. Materials and Methods

### 2.1. Ethics Statement

The study protocol (protocol No. JYNS-CT-CS2025) was reviewed and approved by the Institutional Review Board (IRB) of Woosuk University Korean Medicine Hospital, Jeonju, Republic of Korea (IRB No. WSOH IRB H2509-02; approved 15 September 2025). The trial was retrospectively registered with the Clinical Research Information Service (CRIS) in the Republic of Korea on 27 August 2026, under the identifier KCT0012517 (https://cris.nih.go.kr/cris/index/index.do). Before enrollment, all participants received detailed information on the study objectives, procedures, potential risks, and benefits, and voluntarily provided written informed consent. The study was conducted in accordance with the principles of the Declaration of Helsinki and with the International Council for Harmonisation Good Clinical Practice (ICH-GCP) guidelines, and it is reported in accordance with the Consolidated Standards of Reporting Trials (CONSORT) statement.

### 2.2. Study Participants

Participants with mild knee OA were recruited from Woosuk University Korean Medicine Hospital. Participants were eligible if they met all of the following criteria: (1) age between 45 and 74 years; (2) Kellgren–Lawrence (KL) grade II OA in one or both knees on plain radiography; and (3) knee pain of 40–70 mm on a 100-mm visual analog scale (VAS).

Participants were excluded if they met any of the following criteria: (1) a positive rheumatoid factor (RF) test or suspected rheumatoid arthritis; (2) participation in another clinical trial within 12 weeks before screening; (3) a history of knee surgery or spinal or other lower-extremity joint disorders that could substantially affect the knee; (4) suspected autoimmune disease, inflammatory arthritis, or another musculoskeletal condition that could interfere with the assessment of knee OA; (5) consumption of health functional foods that could affect bone or joint health within 4 weeks before screening; (6) use of medication that could affect study assessments within 12 weeks before screening; (7) habitual use of medications that could affect pain perception within 8 weeks before screening; (8) receipt of acupuncture, physical therapy, or pharmacopuncture for knee symptoms within 4 weeks before study initiation; (9) excessive alcohol consumption (>210 g/week for men or >140 g/week for women); (10) a body mass index (BMI) ≤ 18.5 or >30.0 kg/m^2^; (11) a history or risk of clinically significant hypersensitivity to the study products or their ingredients; (12) clinically significant hepatic, renal, cardiovascular, respiratory, endocrine, neurological, or hematological disorders or malignancy; (13) pregnancy or breastfeeding, or unwillingness of women of childbearing potential to use appropriate contraception; (14) clinically relevant laboratory abnormality at screening; (15) unstable or uncontrolled chronic medical conditions, including diabetes mellitus or hypertension; or (16) any other condition that the principal investigator considered to make the participant unsuitable for study participation.

### 2.3. Study Design and Randomization

This was a 12-week, single-center, randomized, double-blind, placebo-controlled, parallel-group clinical trial conducted at Woosuk University Korean Medicine Hospital. Following the screening period, the 100 eligible participants were randomly assigned in a 1:1 ratio to either the CS group (*n* = 50) or the placebo group (*n* = 50). The randomization sequence was generated before study initiation with R software (version 4.4.3) as a random sequence of treatment codes (A and B), which were assigned sequentially according to participant enrollment number. Both participants and investigators remained blinded to treatment allocation throughout the study period.

Participants completed four study visits: screening (week −2), baseline (week 0), week 6, and week 12. Following randomization at the baseline visit, they took the assigned study product daily for 12 weeks. Efficacy assessments were conducted at baseline and at weeks 6 and 12, except for pain VAS, which was assessed at screening and weeks 6 and 12; the screening value was used as the baseline value for efficacy analyses. A visit window of ±7 days was allowed for the week 6 and week 12 assessments.

Dietary intake and physical activity were assessed at baseline and week 12, so as to evaluate potential changes during the intervention period. Participants were instructed to maintain their usual dietary habits and levels of physical activity throughout the study, and to avoid regular consumption of foods containing bovine bone or cartilage. Dietary intake was assessed from dietary records and analyzed with a Computer-Aided Nutritional Analysis Program 6.0 (CAN-Pro; The Korean Nutrition Society, Seoul, Republic of Korea), and physical activity was assessed with the Global Physical Activity Questionnaire (GPAQ).

### 2.4. Study Products and Interventions

The CS used in this study (batch No. CS9076) was manufactured by Internacional Farmaceutica Argentina S.A. (Buenos Aires, Argentina), a Good Manufacturing Practice (GMP)-compliant manufacturer. The CS was derived from bovine (*Bos taurus*) cartilage and manufactured through enzymatic treatment, filtration, purification, concentration, and drying. The CS was recognized by the Korean Ministry of Food and Drug Safety (MFDS) as an individually recognized functional ingredient (Recognition No. 2020-1) [16], and the batch used in this study complied with the specifications established for the recognized ingredient. The CS was a food-grade material specified to contain not less than 90% chondroitin sulfate sodium on a dry basis [10,17], and the measured content was 97.1%. The chondroitin sulfate sodium content of the CS was determined in accordance with Method 3 for Chondroitin Sulfate specified in the Standards and Specifications for Health Functional Foods issued by the MFDS [18]. The CS was also analyzed for contaminants and found to comply with the specified limits for heavy metals, including lead and arsenic (≤1.0 mg/kg each) and cadmium and mercury (≤0.5 mg/kg each), and was negative for *Escherichia coli*.

For the clinical trial, the study products were provided by Ju Yeong NS (Seoul, Republic of Korea). Each active tablet contained 600 mg of CS, and participants assigned to the CS group consumed two tablets once daily for 12 weeks, giving a total daily dose of 1200 mg of CS.

The placebo was formulated without CS and contained crystalline cellulose as the primary ingredient. Participants in the placebo group consumed two placebo tablets once daily on the same administration schedule as the CS group. Both the CS and placebo products were white tablets of comparable appearance and size, with no apparent differences in taste and no distinctive odor. Therefore, the two products were not readily distinguishable by participants or investigators, thereby maintaining double blinding throughout the study.

Compliance with study product intake was assessed at weeks 6 and 12 from the proportion of the prescribed study product consumed during the intervention period, and participants with an overall compliance rate of ≥ 80% were considered compliant.

### 2.5. Efficacy Outcome Measures

#### 2.5.1. Primary Efficacy Outcomes Measures

The primary efficacy outcomes were the total and subscale scores of the Korean version of the Western Ontario and McMaster Universities Osteoarthritis Index (K-WOMAC) [19], assessed at baseline and at weeks 6 and 12. The K-WOMAC comprises 24 items across three subscales: pain (5 items), stiffness (2 items), and physical function (17 items). Each item was scored on a 5-point scale from 0 to 4, which yields score ranges of 0–20 for pain, 0–8 for stiffness, and 0–68 for physical function, and a total score ranging from 0 to 96. Higher scores indicate greater pain and stiffness and poorer physical function, and changes from baseline in the K-WOMAC total and subscale scores were evaluated throughout the 12-week intervention period.

#### 2.5.2. Secondary Efficacy Outcomes Measures

The secondary efficacy outcomes were knee pain, assessed with a VAS; walking performance, assessed with a 20-m walking test; hand symptoms and function, assessed with the Korean version of the Australian/Canadian Osteoarthritis Hand Index (K-AUSCAN); and hand strength, assessed as grip and pinch strength.

Knee pain was assessed with a 100-mm VAS ranging from 0 mm (no pain) to 100 mm (worst imaginable pain) [20], higher scores indicating greater pain severity. The VAS was administered at screening and at weeks 6 and 12; the screening VAS score served as the baseline value for efficacy analyses and was also used to determine eligibility, participants being required to have a VAS score of 40–70 mm.

Walking performance was assessed at baseline and at weeks 6 and 12 with a 20-m walking test. Participants were instructed to walk a 20-m straight course at their usual walking speed, and the time taken to complete the course was recorded in seconds. The test was performed three consecutive times, and the mean of the three measurements was used for analysis. Participants who required an assistive device were permitted to use it, provided that the same device was used at each assessment.

Hand-related outcomes were included as supportive secondary measures to complement the knee-specific assessments by providing additional information on joint-related symptoms and physical function. The K-AUSCAN was used to assess self-reported hand symptoms and function, whereas grip and pinch strength were included as objective measures of hand function. The K-AUSCAN was administered at baseline and at weeks 6 and 12 to assess hand-related pain, stiffness, and physical function [21]. The questionnaire comprises 15 items across three subscales: pain (5 items), stiffness (1 item), and physical function (9 items). Each item was rated on a 5-point Likert scale from 0 to 4, which yields score ranges of 0–20 for pain, 0–4 for stiffness, and 0–36 for physical function, and a total score ranging from 0 to 60. As for the K-WOMAC, higher scores indicate greater pain and stiffness and poorer physical function.

Grip and pinch strength were measured in both hands at baseline and at weeks 6 and 12. Grip strength was measured in the standing position, with the arms extended downward and the wrist held in a neutral position. Participants exerted maximal force for 3 s, with a 30-s rest between measurements. The dominant hand was assessed first, followed by the non-dominant hand. Three measurements were obtained for each hand, and the highest value was used for analysis.

Pinch strength was measured in the seated position for three pinch types: fingertip, lateral, and three-jaw chuck. Participants exerted maximal force for 3 s, with a 10-s rest between measurements. The dominant hand was assessed first, followed by the non-dominant hand. Each measurement was performed twice, and the highest value was used for analysis.

### 2.6. Safety Outcome Measures

Safety was evaluated throughout the study by monitoring adverse events (AEs) and by performing clinical laboratory tests and vital sign measurements. AEs were identified through non-directive questioning at study visits, spontaneous reports by participants, physical examination, laboratory tests, or other study assessments, and their severity and their causal relationship to the study products were evaluated.

Clinical laboratory tests were performed at screening and at week 12, after fasting for at least 8 h. The laboratory assessments comprised hematological parameters [red blood cell (RBC) count, white blood cell (WBC) count, hemoglobin, hematocrit, platelet count, differential leukocyte count, and erythrocyte sedimentation rate (ESR)], blood biochemical parameters [aspartate aminotransferase (AST), alanine aminotransferase (ALT), gamma-glutamyl transferase (γ-GTP), total protein, albumin, glucose, total bilirubin, electrolytes, creatinine, blood urea nitrogen (BUN), uric acid, and high-sensitivity C-reactive protein (hs-CRP)], and urinalysis. Thyroid-stimulating hormone (TSH) and rheumatoid factor were measured at screening only. Vital signs—systolic blood pressure (SBP), diastolic blood pressure (DBP), pulse rate, and body temperature—were measured at screening and at weeks 6 and 12, after participants had rested for at least 5 min.

### 2.7. Sample Size Calculation

The sample size was calculated from the expected difference in the change in K-WOMAC total score between the CS and placebo groups. The power calculation was performed with reference to a previous randomized controlled study by Park et al. [22], which reported a between-group difference of 8.02 points and a pooled standard deviation of 12.61. Assuming a two-sided significance level of 0.05, 80% statistical power, and a 1:1 allocation ratio, a minimum of 39 participants per group was required. Considering an anticipated dropout rate of 20%, the target sample size was set at 100 participants, with 50 participants assigned to each group.

### 2.8. Statistical Analysis

All statistical analyses were performed with IBM SPSS Statistics (version 32.0; IBM Corp., Armonk, NY, USA). Continuous variables are presented as means ± standard deviations (SD) and categorical variables as numbers and percentages. Efficacy analyses were performed primarily on the per-protocol set (PPS), and the full analysis set (FAS) was analyzed in addition. The FAS included randomized participants who consumed the study product at least once, underwent at least one post-intervention efficacy assessment, and had no major eligibility violations. Any missing post-intervention efficacy data were handled by the last observation carried forward (LOCF) method, and baseline values were not carried forward. The PPS included participants in the FAS who completed the study without major protocol deviations that could affect the efficacy evaluation. Safety analyses were performed using the safety set, which included all randomized participants who consumed the study product at least once.

Within-group changes from baseline were assessed with paired *t*-tests where the normality assumption was met and with the Wilcoxon signed-rank test where the data were not normally distributed. Between-group differences in change from baseline were assessed with independent-samples *t*-tests or the Wilcoxon rank-sum test, as appropriate, and differences between groups in categorical variables were evaluated with the chi-square test or Fisher’s exact test, as appropriate.

Analysis of covariance (ANCOVA) was performed with the relevant baseline characteristics as covariates when statistically significant differences between groups were identified at baseline.

All statistical tests were two-sided, and a *p*-value < 0.05 was considered statistically significant.

## 3. Results

### 3.1. Participant Flow and Baseline Characteristics

**Figure 1:**
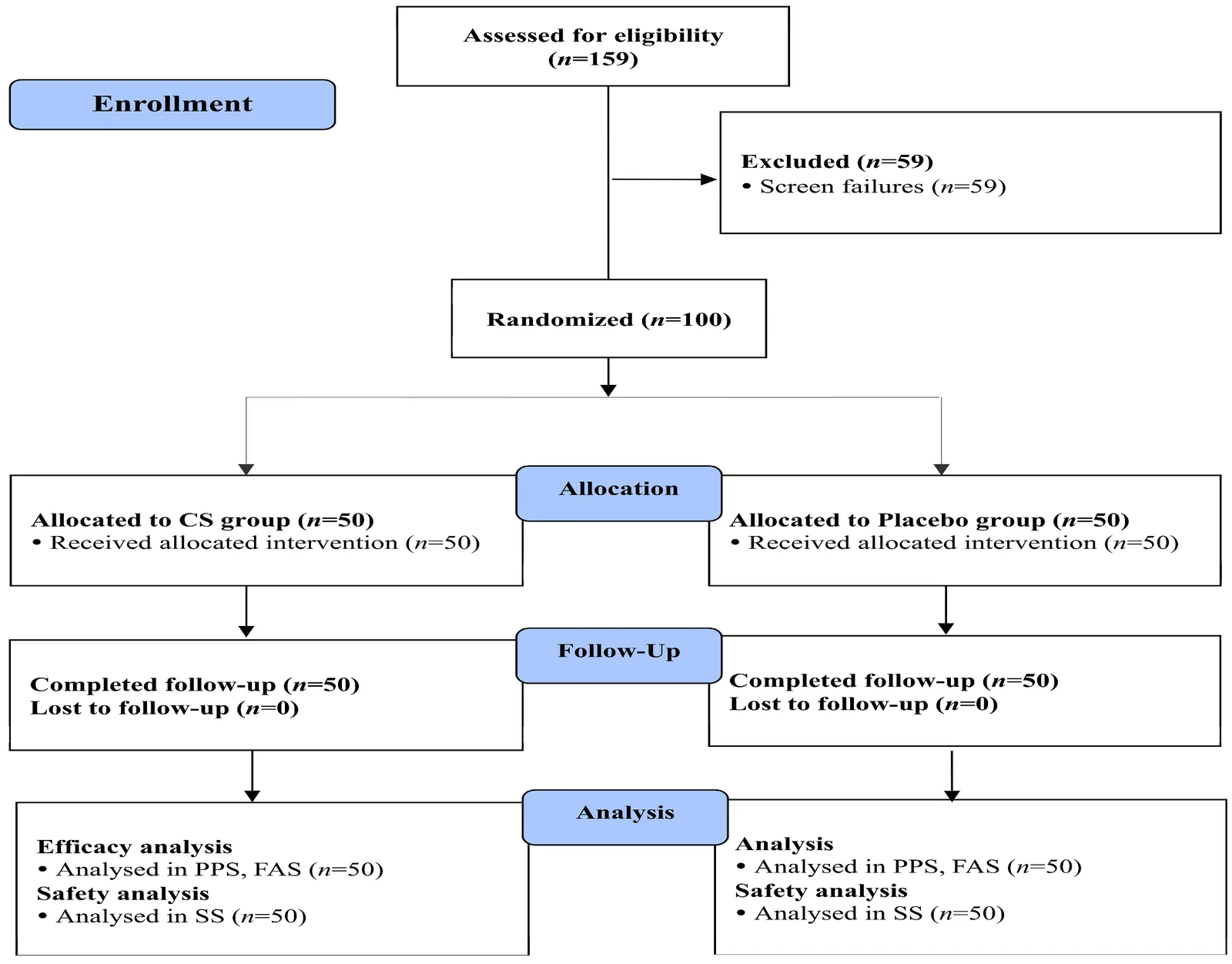
CONSORT flow diagram of study participants.

A total of 159 individuals were screened for eligibility at Woosuk University Korean Medicine Hospital, of whom 59 were excluded during screening; the 100 eligible participants were enrolled and randomly assigned in a 1:1 ratio to either the CS group (*n* = 50) or the placebo group (*n* = 50). All 100 randomized participants completed the 12-week intervention, with no discontinuations. No participant was excluded from the efficacy analysis because of insufficient compliance with the study product or of major protocol violation. Mean compliance with the study product was 99.08 ± 1.04% in the CS group and 98.62 ± 1.25% in the placebo group, with no significant between-group difference (*p* = 0.061). All participants in both groups achieved a compliance rate of ≥80%. Accordingly, the PPS and FAS comprised the same 100 participants (50 per group). Participant enrollment, allocation, follow-up, and analysis are summarized in the CONSORT flow diagram (Figure 1).

Baseline demographic and anthropometric characteristics are presented in Table 1. The mean age was 61.04 ± 4.89 years in the CS group and 61.84 ± 5.13 years in the placebo group. No statistically significant between-group differences were observed at baseline in age, sex distribution, height, body weight, or BMI (all *p* > 0.05), which indicates that the two groups were comparable in their baseline demographic and anthropometric characteristics.

Dietary intake and physical activity were also assessed, so as to determine whether lifestyle-related factors changed during the intervention. No significant between-group differences were observed in dietary intake or physical activity at baseline or at week 12, and the changes from baseline did not differ significantly between the CS and placebo groups (all *p* > 0.05), which indicates that dietary intake and physical activity remained comparable between the groups throughout the intervention period.

### 3.2. Primary Efficacy Outcomes: K-WOMAC Scores

**Figure 2:**
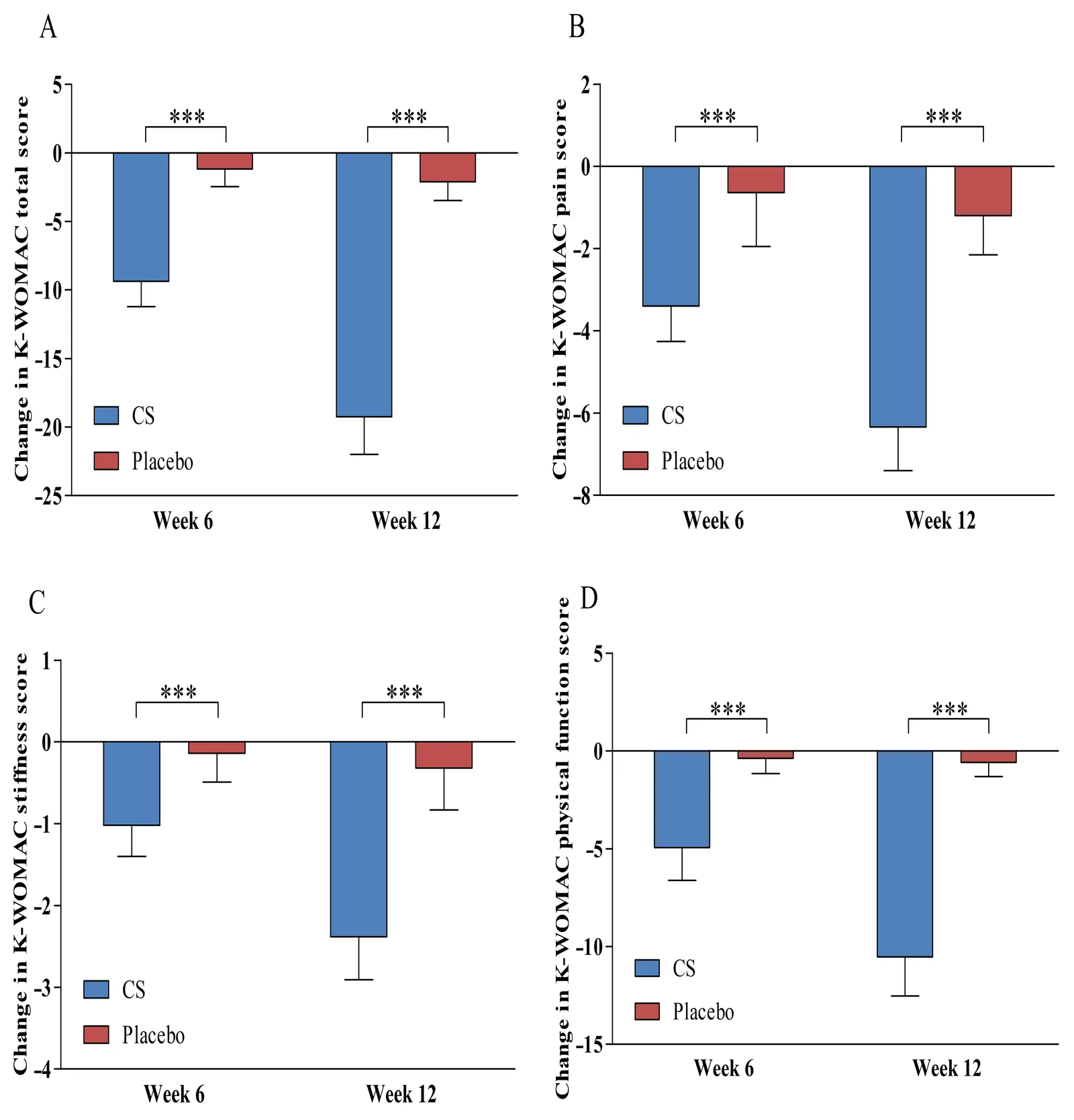
Changes in K-WOMAC total and subscale scores after the 12-week intervention. (**A**) K-WOMAC total score, (**B**) pain score, (**C**) stiffness score, and (**D**) physical function score in the CS and placebo groups. Values are presented as mean ± SD. Between-group differences in changes from baseline were analyzed using the Wilcoxon rank-sum test. For K-WOMAC total and pain scores, ANCOVA adjusted for the corresponding baseline values was additionally performed due to significant between-group differences at baseline. *** *p* < 0.001 vs. placebo group.

Changes in the K-WOMAC total and subscale scores during the 12-week intervention are presented in Table 2 and illustrated in Figure 2. At baseline, the K-WOMAC total score was 38.70 ± 3.80 in the CS group and 37.14 ± 4.67 in the placebo group (*p* = 0.029). At week 6, the total score had decreased by 9.36 ± 1.85 points in the CS group and by 1.16 ± 1.23 points in the placebo group, and at week 12 the reductions were 19.26 ± 2.74 and 2.10 ± 1.37 points, respectively. The reductions from baseline were significantly greater in the CS group than in the placebo group at both week 6 and week 12 (both *p* < 0.001). Because the baseline K-WOMAC total score differed significantly between the groups, ANCOVA was performed with the baseline value as a covariate, and the between-group differences remained significant after adjustment (both *p* < 0.001).

Similar patterns were observed for the K-WOMAC subscale scores: the reductions from baseline in pain, stiffness, and physical function were significantly greater in the CS group than in the placebo group at both week 6 and week 12 (all *p* < 0.001). For the pain subscale, which differed significantly between groups at baseline (*p* = 0.021), the between-group differences remained significant after adjustment for the baseline value using ANCOVA (both *p* < 0.001).

### 3.3. Secondary Efficacy Outcomes

#### 3.3.1. Pain VAS and Walking Performance

**Figure 3:**
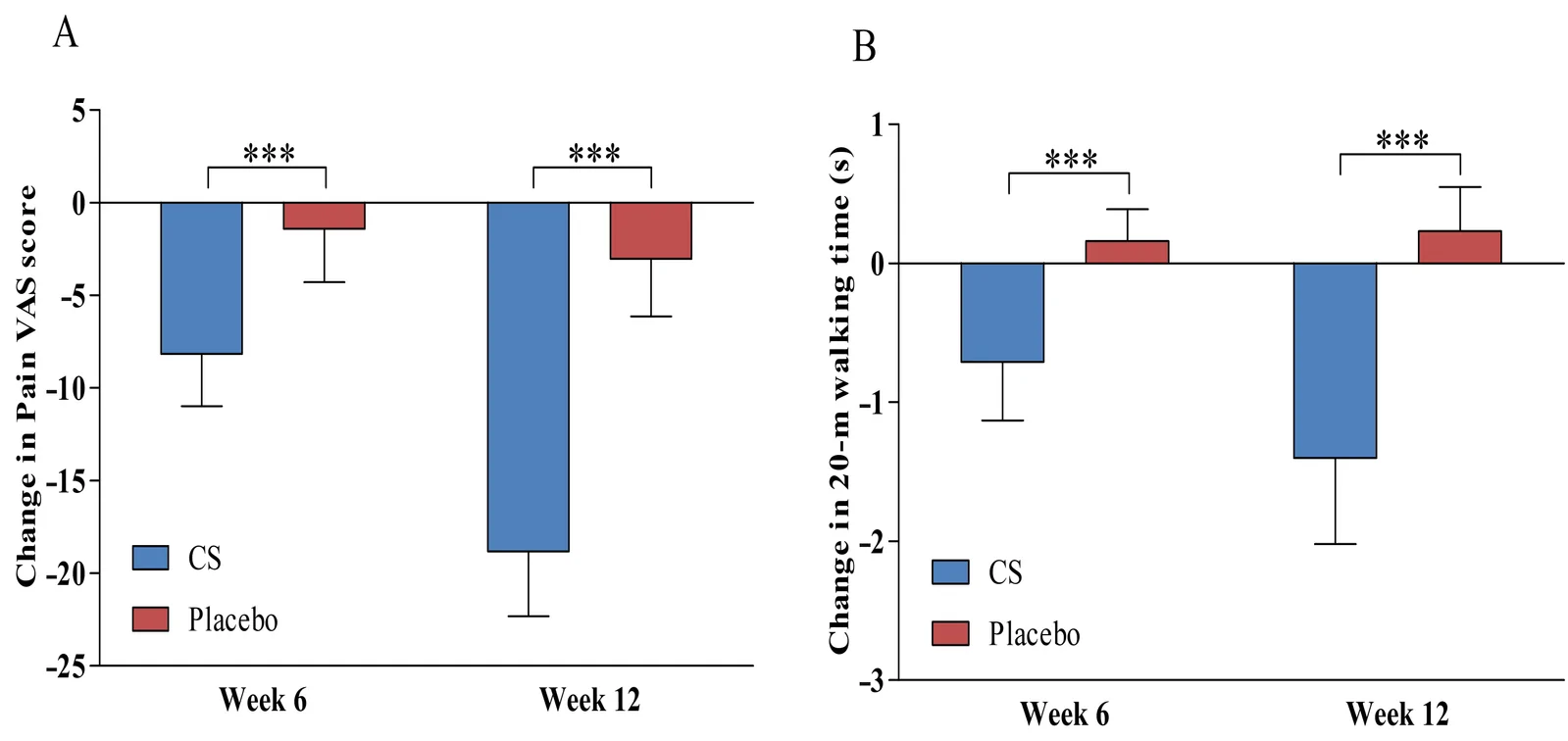
Changes in pain VAS and 20-m walking time after the 12-week intervention. (**A**) Pain VAS score and (**B**) 20-m walking time in the CS and placebo groups. Values are presented as mean ± SD. Between-group differences in changes from baseline were analyzed using the Wilcoxon rank-sum test. *** *p* < 0.001 vs. placebo group.

Changes in pain VAS and in 20-m walking time during the 12-week intervention are presented in Table 3 and illustrated in Figure 3. Pain VAS scores decreased from baseline by 8.16 ± 2.82 mm at week 6 and by 18.82 ± 3.50 mm at week 12 in the CS group, compared with decreases of 1.42 ± 2.88 mm and 3.04 ± 3.10 mm, respectively, in the placebo group. The reductions were significantly greater in the CS group than in the placebo group at both time points (both *p* < 0.001).

The 20-m walking time similarly decreased by 0.71 ± 0.42 s at week 6 and by 1.40 ± 0.62 s at week 12 in the CS group, whereas it increased by 0.16 ± 0.23 s and 0.23 ± 0.32 s, respectively, in the placebo group. The between-group differences in changes from baseline were significant at both time points (both *p* < 0.001).

#### 3.3.2. K-AUSCAN Scores

Changes in the K-AUSCAN total and subscale scores during the 12-week intervention are presented in Table 4. The K-AUSCAN total score decreased by 7.96 ± 1.73 points at week 6 and by 16.74 ± 2.50 points at week 12 in the CS group, compared with decreases of 0.38 ± 1.10 and 1.08 ± 1.26 points, respectively, in the placebo group. At both time points, the reductions were significantly greater in the CS group than in the placebo group (both *p* < 0.001).

Consistent with the total score, the reductions in the K-AUSCAN pain, stiffness, and physical function subscale scores were significantly greater in the CS group than in the placebo group at both week 6 and week 12 (all *p* < 0.001).

#### 3.3.3. Grip and Pinch Strength

Changes in grip and pinch strength during the 12-week intervention are presented in Table 5. Grip strength increased in both hands in the CS group, whereas it decreased in the placebo group, and the changes from baseline in both right- and left-hand grip strength differed significantly between the groups at weeks 6 and 12 (all *p* < 0.001).

Fingertip, lateral, and three-jaw chuck pinch strength likewise increased in both hands in the CS group, whereas these measures generally decreased in the placebo group. For all pinch strength measures, the changes from baseline differed significantly between the groups at both week 6 and week 12 (all *p* < 0.001). For measures with significant baseline differences, the between-group differences remained significant after adjustment for baseline values using ANCOVA (all adjusted *p* < 0.001).

### 3.4. Safety Outcomes

Safety analyses were performed using the safety set. No adverse events (AEs) were reported in either group during the 12-week intervention. Clinical laboratory assessments, comprising hematological, biochemical, and urinalysis parameters, showed no clinically meaningful change from screening to week 12. Detailed hematological and biochemical results are provided in Table S1. Vital signs—systolic and diastolic blood pressure, pulse rate, and body temperature—remained clinically stable throughout the study (Table S2). Overall, CS supplementation was well tolerated over the 12-week intervention period.

## 4. Discussion

This randomized, double-blind, placebo-controlled trial showed that 12 weeks of supplementation with CS at 1200 mg/day improved joint symptoms and multiple aspects of physical function in adults with mild knee OA. Relative to placebo, CS supplementation produced greater reductions in the K-WOMAC total score and in its pain, stiffness, and physical function subscale scores. Consistent improvements were also observed in pain VAS and 20-m walking time, and K-AUSCAN scores decreased while grip and pinch strength increased following CS supplementation. Improvements in self-reported symptoms were notably accompanied by favorable changes in performance- and strength-based measures. These findings suggest that the potential benefits of CS in adults with mild knee OA may extend beyond symptom relief to broader aspects of physical function. Overall, CS supplementation was well tolerated during the 12-week intervention.

An important feature of the present study was the evaluation of CS in a well-defined population with relatively mild radiographic knee OA. Participants were restricted to radiographically confirmed KL grade II OA and were additionally required to have clinically relevant knee pain (VAS, 40–70 mm). Previous CS trials have included broader populations spanning multiple radiographic grades and levels of symptom severity [13,23,24,25]. By restricting enrollment to KL grade II while requiring a defined range of knee pain, the present study provided a relatively homogeneous population with respect to radiographic severity and symptom burden. This is clinically relevant, because radiographic severity does not fully reflect the burden of pain and functional limitation experienced by individuals with knee OA [26,27]. The present findings therefore provide additional evidence for the symptomatic and functional effects of CS in a clearly characterized population with relatively mild radiographic knee OA.

Another notable aspect of the present study was the consistency of the improvement across complementary measures of joint symptoms and physical function. Previous clinical trials of CS have reported improvements primarily in pain and in composite functional indices such as the VAS, WOMAC, and LI, although the magnitude and consistency of these effects have varied across studies [11,12,13,14,23,24,25]. In the present study, CS supplementation produced greater reductions in the K-WOMAC total score and in pain VAS over 12 weeks, the improvements being already evident at week 6 and more pronounced by week 12. These findings are consistent with those of Zegels et al., who reported improvements in pain and the LI after 3 months of CS supplementation at 1200 mg/day [11], and with other placebo-controlled trials demonstrating symptomatic and functional benefits of CS [12,23,24]. The improvements in self-reported outcomes in the present study were moreover accompanied by a significant reduction in 20-m walking time relative to placebo. The between-group difference in walking time was apparent from week 6 and was maintained through week 12. Self-reported and performance-based measures capture distinct but complementary aspects of physical function in individuals with knee OA [28]. Whereas the K-WOMAC reflects perceived pain, stiffness, and functional limitation, timed walking assesses mobility directly, through actual physical performance [28]. The 20-m walk has also been used as an objective measure of habitual walking performance in individuals with or at risk of knee OA. Previous randomized controlled trials of CS in knee OA have also evaluated 20-m walking time, and reported significant between-group differences after 6 months of treatment or follow-up [12,15], whereas in the present study a significant between-group difference was already apparent at week 6. Direct comparisons across studies should be made cautiously, given the differences in study populations, CS regimens, and designs, but these findings suggest that improvements in objective walking performance may be detectable within a relatively short period of CS supplementation.

Additional improvements were observed in K-AUSCAN scores and measures of grip and pinch strength. CS supplementation produced greater reductions than placebo in the K-AUSCAN total, pain, stiffness, and physical function scores, together with increases in bilateral grip strength and in fingertip, lateral, and three-jaw chuck pinch strength. Previous evidence on the effects of CS on hand symptoms and function is limited. In a randomized, double-blind, placebo-controlled trial in individuals with symptomatic hand OA, Gabay et al. reported that 6 months of CS supplementation significantly improved hand pain and functional impairment relative to placebo, although the between-group difference in grip strength was not significant [29]. In the present study, the parallel improvements in K-AUSCAN scores and in directly measured grip and pinch strength provide supportive evidence that changes in self-reported hand symptoms and functional limitation were accompanied by improvements in measured hand strength. The observed increases in grip and pinch strength should be interpreted cautiously. Although these measures provide objective information on hand performance, the present study was not designed to determine whether CS has a direct effect on skeletal muscle strength. Therefore, the improvements in hand strength are better interpreted as supportive functional findings occurring alongside improvements in self-reported hand symptoms and function, rather than as evidence of a generalized muscle-strengthening effect of CS. These findings should also be interpreted in light of the fact that participants were recruited on the basis of knee OA and were not selected for the presence of hand OA. The K-AUSCAN and hand-strength outcomes should accordingly be regarded as supportive secondary findings rather than as evidence of the efficacy of CS for hand OA.

Several biological actions of CS may provide a plausible basis for the clinical improvements observed here. CS is a major glycosaminoglycan component of the cartilage extracellular matrix, and experimental studies suggest that its effects may extend beyond this structural role to the modulation of the anabolic, catabolic, and inflammatory processes involved in OA [8,30]. CS has in particular been reported to promote the synthesis of cartilage matrix components such as proteoglycans, while reducing the production or activity of the proteolytic enzymes and inflammatory mediators that contribute to cartilage matrix degradation [8,30,31]. Of particular relevance to the present study, preclinical evaluation of the CS preparation used in this trial demonstrated modulation of MMPs and TIMP-1 in a monosodium iodoacetate-induced OA model [10]. These findings provide biological plausibility for the improvements in joint symptoms and physical function observed following CS supplementation. However, the clinical outcomes reported in the present study primarily reflect changes in joint symptoms and physical function. The observed clinical improvements should therefore not be interpreted as evidence of a cartilage-protective or disease-modifying effect, and the mechanisms underlying them remain to be established.

The present study has several strengths. First, the randomized, double-blind, placebo-controlled design minimized potential selection and assessment bias, and all randomized participants completed the 12-week intervention without major protocol violation. Second, the inclusion of participants with radiographically confirmed KL grade II knee OA and a defined range of clinically relevant knee pain provided a well-characterized study population of relatively homogeneous radiographic severity. Third, clinical response was evaluated across multiple complementary outcome domains, including knee-specific symptoms and function, pain intensity, performance-based mobility, and strength measures. This multidimensional approach is particularly relevant in knee OA, because self-reported and performance-based assessments capture related but distinct aspects of physical function [28]. The consistency of the improvements across these different outcome domains strengthens the overall interpretation of the efficacy findings.

From a sports nutrition perspective, the present findings should not be extrapolated to athletic performance or sports supplementation. Chondroitin sulfate is not currently recognized as an evidence-based ergogenic supplement within major sports nutrition frameworks, and evidence supporting its use for enhancing exercise performance or recovery remains limited. Accordingly, the present findings should be interpreted specifically in the context of symptom and physical function management in adults with knee OA rather than as evidence supporting CS supplementation for athletic performance.

Several limitations should be considered when interpreting the present findings. First, this was a single-center study in a relatively homogeneous and selected population with KL grade II knee OA, with restrictions related to BMI, comorbidities, concomitant treatments, and symptom severity, as well as a predominance of female participants. These characteristics may limit the generalizability of the findings to the broader knee OA population. Second, the 12-week intervention period was appropriate for evaluating changes in joint symptoms and physical function but was insufficient to determine the durability of these effects or to detect potential structural change. Moreover, imaging modalities such as magnetic resonance imaging (MRI) or ultrasonography were not included, precluding direct assessment of structural changes in the knee joint. Previous long-term CS trials evaluating structural outcomes have used substantially longer follow-up periods, including 2-year assessments of radiographic joint-space narrowing [25,32]. The present findings should therefore be interpreted as evidence of short-term symptomatic and functional improvement rather than of structural or disease-modifying effects. Third, although improvements were observed across multiple outcome domains, the K-AUSCAN and hand-strength measures were secondary outcomes in participants recruited on the basis of knee rather than hand OA, and should therefore be interpreted as supportive findings. Fourth, the trial was registered retrospectively after participant enrollment had begun. Although the study protocol received IRB approval before enrollment, retrospective registration limits prospective public verification of the prespecified study information.

Within these limitations, the present study provides additional evidence that CS supplementation may improve joint symptoms and physical function in adults with mild knee OA. The consistency of the findings across self-reported symptoms and objective performance-based measures further supports the relevance of evaluating multiple complementary functional outcomes when assessing the effects of CS supplementation.

## 5. Conclusions

This randomized, double-blind, placebo-controlled trial demonstrated that 12 weeks of CS supplementation at 1200 mg/day improved joint symptoms and physical function in adults with mild knee OA. In a well-defined population with KL grade II knee OA and clinically relevant knee pain, CS supplementation resulted in improvements in K-WOMAC and pain VAS, accompanied by favorable changes in walking performance and in additional functional measures. The consistency of these findings across self-reported symptoms and objective performance-based outcomes suggests that the effects of CS may extend beyond pain relief to multiple aspects of physical function, and CS supplementation was well tolerated throughout the intervention. Given the 12-week intervention period, these findings should not be interpreted as evidence of structural or disease-modifying effects of CS. Overall, these findings support a potential role for CS as a nutritional approach to improving joint symptoms and physical function in adults with mild knee OA.

## Figures and Tables

**Table 1:** Baseline characteristics of the study participants.

Variables	CS Group (*n* = 50)	Placebo Group (*n* = 50)	*p*-Value ^(1)^
Age (years)	61.04 ± 4.89	61.84 ± 5.13	0.426
Sex (M/F)	5/45	7/43	0.538 ^(2)^
Height (cm)	159.43 ± 6.90	159.40 ± 6.93	0.948 ^(3)^
Weight (kg)	63.44 ± 10.45	61.53 ± 9.65	0.270 ^(3)^
BMI (kg/m^2^)	24.82 ± 2.58	24.11 ± 2.45	0.119 ^(3)^

Values are presented as mean ± SD or N; ^(1)^ analyzed by independent *t*-test between the groups; ^(2)^ analyzed by chi-squared test between the groups; ^(3)^ analyzed by Wilcoxon rank-sum test between the groups. M, male; F, female; BMI, body mass index.

**Table 2:** Changes in K-WOMAC total and subscale scores during the 12-week intervention.

Variables		CS Group (*n* = 50)	Placebo Group (*n* = 50)	*p*-Value ^(1)^
Total score	Baseline	38.70 ± 3.80	37.14 ± 4.67	0.029
	Week 6	29.34 ± 3.40	35.98 ± 4.48	<0.001 (<0.001) ^(3)^
	Change from baseline	−9.36 ± 1.85	−1.16 ± 1.23	<0.001 (<0.001) ^(3)^
	*p*-value ^(2)^	<0.001	<0.001	
	Week 12	19.44 ± 3.10	35.04 ± 4.15	<0.001 (<0.001) ^(3)^
	Change from baseline	−19.26 ± 2.74	−2.10 ± 1.37	<0.001 (<0.001) ^(3)^
	*p*-value ^(2)^	<0.001	<0.001	
Pain	Baseline	9.68 ± 1.32	9.08 ± 1.37	0.021
	Week 6	6.28 ± 1.20	8.44 ± 1.34	<0.001 (<0.001) ^(3)^
	Change from baseline	−3.40 ± 0.86	−0.64 ± 0.66	<0.001 (<0.001) ^(3)^
	*p*-value ^(2)^	<0.001	<0.001	
	Week 12	3.34 ± 0.94	7.88 ± 1.22	<0.001 (<0.001) ^(3)^
	Change from baseline	−6.34 ± 1.06	−1.20 ± 0.95	<0.001 (<0.001) ^(3)^
	*p*-value ^(2)^	<0.001	<0.001	
Stiffness	Baseline	4.18 ± 0.48	4.20 ± 0.57	0.930
	Week 6	3.16 ± 0.62	4.06 ± 0.62	<0.001
	Change from baseline	−1.02 ± 0.38	−0.14 ± 0.35	<0.001
	*p*-value ^(2)^	<0.001	0.008	
	Week 12	1.80 ± 0.45	3.88 ± 0.56	<0.001
	Change from baseline	−2.38 ± 0.53	−0.32 ± 0.51	<0.001
	*p*-value ^(2)^	<0.001	<0.001	
Physical function	Baseline	24.84 ± 2.61	23.86 ± 3.28	0.068
	Week 6	19.90 ± 2.23	23.48 ± 3.18	<0.001
	Change from baseline	−4.94 ± 1.68	−0.38 ± 0.78	<0.001
	*p*-value ^(2)^	<0.001	0.001	
	Week 12	14.30 ± 2.23	23.28 ± 3.17	<0.001
	Change from baseline	−10.54 ± 1.99	−0.58 ± 0.73	<0.001
	*p*-value ^(2)^	<0.001	<0.001	

Values are presented as mean ± SD; ^(1)^ analyzed by Wilcoxon rank-sum test between the groups; ^(2)^ analyzed by Wilcoxon signed-rank test within each group; ^(3)^ analyzed by ANCOVA (adjusted for baseline values). K-WOMAC, Korean Western Ontario and McMaster Universities Osteoarthritis Index; ANCOVA, analysis of covariance.

**Table 3:** Changes in pain VAS and 20-m walking time during the 12-week intervention.

Variables		CS Group (*n* = 50)	Placebo Group (*n* = 50)	*p*-Value ^(1)^
Pain VAS	Baseline	55.60 ± 2.97	54.50 ± 3.54	0.096
	Week 6	47.44 ± 3.13	53.08 ± 3.83	<0.001
	Change from baseline	−8.16 ± 2.82	−1.42 ± 2.88	<0.001
	*p*-value ^(2)^	<0.001 ^(3)^	0.003 ^(3)^	
	Week 12	36.78 ± 3.30	51.46 ± 3.86	<0.001
	Change from baseline	−18.82 ± 3.50	−3.04 ± 3.10	<0.001
	*p*-value ^(2)^	<0.001 ^(3)^	<0.001 ^(3)^	
20-m walking time (s)	Baseline	13.79 ± 1.42	14.14 ± 1.78	0.497
	Week 6	13.08 ± 1.36	14.30 ± 1.89	0.002
	Change from baseline	−0.71 ± 0.42	0.16 ± 0.23	<0.001
	*p*-value ^(2)^	<0.001 ^(3)^	<0.001 ^(3)^	
	Week 12	12.39 ± 1.16	14.38 ± 1.88	<0.001
	Change from baseline	−1.40 ± 0.62	0.23 ± 0.32	<0.001
	*p*-value ^(2)^	<0.001 ^(3)^	<0.001	

Values are presented as mean ± SD; ^(1)^ analyzed by Wilcoxon rank-sum test between the groups; ^(2)^ analyzed by paired *t*-test within each group; ^(3)^ analyzed by Wilcoxon signed-rank test within each group. VAS, visual analog scale.

**Table 4:** Changes in K-AUSCAN total and subscale scores during the 12-week intervention.

Variables		CS Group (*n* = 50)	Placebo Group (*n* = 50)	*p*-Value ^(1)^
K-AUSCAN Total Score	Baseline	33.54 ± 3.64	32.34 ± 4.56	0.149
	Week 6	25.58 ± 3.28	31.96 ± 4.27	<0.001
	Change from baseline	−7.96 ± 1.73	−0.38 ± 1.10	<0.001 ^(2)^
	*p*-value ^(3)^	<0.001 ^(4)^	0.003 ^(4)^	
	Week 12	16.80 ± 2.47	31.26 ± 4.16	<0.001
	Change from baseline	−16.74 ± 2.50	−1.08 ± 1.26	<0.001 ^(2)^
	*p*-value ^(3)^	<0.001	<0.001 ^(4)^	
K-AUSCAN Pain	Baseline	11.08 ± 1.29	10.58 ± 1.55	0.056 ^(2)^
	Week 6	7.64 ± 1.21	10.44 ± 1.43	<0.001 ^(2)^
	Change from baseline	−3.44 ± 0.73	−0.14 ± 0.57	<0.001 ^(2)^
	*p*-value ^(3)^	<0.001 ^(4)^	0.097 ^(4)^	
	Week 12	4.36 ± 0.98	10.10 ± 1.37	<0.001 ^(2)^
	Change from baseline	−6.72 ± 1.21	−0.48 ± 0.79	<0.001 ^(2)^
	*p*-value ^(3)^	<0.001 ^(4)^	<0.001 ^(4)^	
K-AUSCAN Stiffness	Baseline	2.30 ± 0.51	2.30 ± 0.46	0.949 ^(2)^
	Week 6	1.52 ± 0.50	2.18 ± 0.44	<0.001 ^(2)^
	Change from baseline	−0.78 ± 0.42	−0.12 ± 0.33	<0.001 ^(2)^
	*p*-value ^(3)^	<0.001 ^(4)^	0.014 ^(4)^	
	Week 12	0.84 ± 0.37	2.12 ± 0.39	<0.001 ^(2)^
	Change from baseline	−1.46 ± 0.50	−0.18 ± 0.39	<0.001 ^(2)^
	*p*-value ^(3)^	<0.001 ^(4)^	0.003 ^(4)^	
K-AUSCAN Physical Function	Baseline	20.16 ± 2.57	19.46 ± 2.98	0.272 ^(2)^
	Week 6	16.42 ± 2.34	19.34 ± 2.89	<0.001 ^(2)^
	Change from baseline	−3.74 ± 1.23	−0.12 ± 0.66	<0.001 ^(2)^
	*p*-value ^(3)^	<0.001 ^(4)^	0.107 ^(4)^	
	Week 12	11.60 ± 1.74	19.04 ± 2.87	<0.001 ^(2)^
	Change from baseline	−8.56 ± 1.74	−0.42 ± 0.73	<0.001 ^(2)^
	*p*-value ^(3)^	<0.001	<0.001 ^(4)^	

Values are presented as mean ± SD; ^(1)^ analyzed by independent *t*-test between the groups; ^(2)^ analyzed by Wilcoxon rank-sum test between the groups; ^(3)^ analyzed by paired *t*-test within each group; ^(4)^ analyzed by Wilcoxon signed-rank test within each group. K-AUSCAN, Korean version of the Australian/Canadian Osteoarthritis Hand Index.

**Table 5:** Changes in grip and pinch strength during the 12-week intervention.

Variables		CS Group (*n* = 50)	Placebo Group (*n* = 50)	*p*-Value ^(1)^
Grip strength, right (kg)	Baseline	23.06 ± 5.93	24.07 ± 5.92	0.504
	Week 6	23.75 ± 5.95	23.43 ± 5.65	0.424
	Change from baseline	0.69 ± 0.91	−0.64 ± 0.67	<0.001
	*p*-value ^(2)^	<0.001 ^(3)^	<0.001 ^(3)^	
	Week 12	24.48 ± 5.72	22.99 ± 5.51	0.026
	Change from baseline	1.43 ± 1.17	−1.08 ± 1.07	<0.001
	*p*-value ^(2)^	<0.001 ^(3)^	<0.001 ^(3)^	
Grip strength, left (kg)	Baseline	21.29 ± 5.48	22.49 ± 6.25	0.664
	Week 6	21.97 ± 5.52	21.97 ± 6.08	0.456
	Change from baseline	0.68 ± 0.91	−0.52 ± 0.77	<0.001
	*p*-value ^(2)^	<0.001 ^(3)^	<0.001	
	Week 12	22.91 ± 5.05	21.79 ± 5.94	0.051
	Change from baseline	1.62 ± 1.36	−0.70 ± 1.01	<0.001
	*p*-value ^(2)^	<0.001 ^(3)^	<0.001	
Fingertip pinch strength, right (kg)	Baseline	4.94 ± 1.26	5.54 ± 1.32	0.010
	Week 6	5.64 ± 1.19	5.25 ± 1.10	0.145 (<0.001) ^(4)^
	Change from baseline	0.70 ± 0.67	−0.29 ± 0.44	<0.001 (<0.001) ^(4)^
	*p*-value ^(2)^	<0.001 ^(3)^	<0.001 ^(3)^	
	Week 12	6.16 ± 1.06	5.14 ± 1.03	<0.001 (<0.001) ^(4)^
	Change from baseline	1.22 ± 0.78	−0.40 ± 0.52	<0.001 (<0.001) ^(4)^
	*p*-value ^(2)^	<0.001 ^(3)^	<0.001 ^(3)^	
Fingertip pinch strength, left (kg)	Baseline	4.55 ± 1.20	5.15 ± 1.19	0.010
	Week 6	5.14 ± 1.15	4.97 ± 1.09	0.486 (<0.001) ^(4)^
	Change from baseline	0.59 ± 0.43	−0.18 ± 0.40	<0.001 (<0.001) ^(4)^
	*p*-value ^(2)^	<0.001 ^(3)^	0.002 ^(3)^	
	Week 12	5.68 ± 1.02	4.99 ± 1.00	<0.001 (<0.001) ^(4)^
	Change from baseline	1.13 ± 0.50	−0.16 ± 0.53	<0.001 (<0.001) ^(4)^
	*p*-value ^(2)^	<0.001 ^(3)^	0.035 ^(3)^	
Lateral pinch strength, right (kg)	Baseline	6.74 ± 1.67	7.05 ± 1.54	0.131
	Week 6	7.02 ± 1.73	6.68 ± 1.53	0.406
	Change from baseline	0.28 ± 0.38	−0.37 ± 0.35	<0.001
	*p*-value ^(2)^	<0.001 ^(3)^	<0.001 ^(3)^	
	Week 12	7.33 ± 1.66	6.46 ± 1.45	0.009
	Change from baseline	0.59 ± 0.45	−0.59 ± 0.42	<0.001
	*p*-value ^(2)^	<0.001 ^(3)^	<0.001 ^(3)^	
Lateral pinch strength, left (kg)	Baseline	6.10 ± 1.56	6.61 ± 1.45	0.035
	Week 6	6.42 ± 1.58	6.32 ± 1.32	0.953 (<0.001) ^(4)^
	Change from baseline	0.32 ± 0.33	−0.29 ± 0.32	<0.001 (<0.001) ^(4)^
	*p*-value ^(2)^	<0.001 ^(3)^	<0.001 ^(3)^	
	Week 12	6.70 ± 1.48	6.06 ± 1.26	0.038 (<0.001) ^(4)^
	Change from baseline	0.60 ± 0.44	−0.55 ± 0.43	<0.001 (<0.001) ^(4)^
	*p*-value ^(2)^	<0.001 ^(3)^	<0.001 ^(3)^	
Three-jaw chuck pinch strength, right (kg)	Baseline	6.05 ± 1.22	6.40 ± 1.40	0.141
	Week 6	6.50 ± 1.22	6.22 ± 1.36	0.284
	Change from baseline	0.45 ± 0.38	−0.18 ± 0.33	<0.001
	*p*-value ^(2)^	<0.001 ^(3)^	<0.001 ^(3)^	
	Week 12	6.90 ± 1.16	6.12 ± 1.37	0.002
	Change from baseline	0.85 ± 0.51	−0.28 ± 0.43	<0.001
	*p*-value ^(2)^	<0.001 ^(3)^	<0.001 ^(3)^	
Three-jaw chuck pinch strength, left (kg)	Baseline	5.73 ± 1.26	6.05 ± 1.37	0.290
	Week 6	6.17 ± 1.22	5.94 ± 1.28	0.282
	Change from baseline	0.44 ± 0.41	−0.11 ± 0.25	<0.001
	*p*-value ^(2)^	<0.001 ^(3)^	0.005 ^(3)^	
	Week 12	6.54 ± 1.21	5.82 ± 1.27	0.003
	Change from baseline	0.81 ± 0.51	−0.23 ± 0.35	<0.001
	*p*-value ^(2)^	<0.001 ^(3)^	<0.001 ^(3)^	

Values are presented as mean ± SD; ^(1)^ analyzed by Wilcoxon rank-sum test between the groups; ^(2)^ analyzed by paired *t*-test within each group; ^(3)^ analyzed by Wilcoxon signed-rank test within each group; ^(4)^ analyzed by ANCOVA (adjusted for baseline values). ANCOVA, analysis of covariance.

## Data Availability

The data presented in this study are available on request from the corresponding author due to privacy and ethical considerations related to participant information.

## Supplementary Materials

The following supporting information can be downloaded at https://www.mdpi.com/article/10.3390/nu18203391/s1: Table S1: Changes in hematological and biochemical parameters from baseline to week 12. Table S2. Changes in vital signs during the 12-week intervention.
