# Supplementary material for: Effects of Chondroitin Sulfate on Joint Symptoms and Physical Function in Adults with Mild Knee Osteoarthritis: A Randomized, Double-Blind, Placebo-Controlled Trial
Source: Nutrients. 2026 Oct 16;18(20):3391. doi: 10.3390/nu18203391 (PMC13708518; doi:10.3390/nu18203391)
Supplement: Supplementary file 1 [file nutrients-18-03391-s001.zip › nutrients-4587120-supplementary.pdf]

Table S1: Changes in hematological and biochemical parameters from baseline to week 12.

Variables		CS Group (*n*=50)		Placebo Group (*n*=50)		*p*-value [(1)]
		**Baseline**	**Week 12**	**Baseline**	**Week 12**	
Hematological parameters	RBC (10^6/μL)	4.55 ± 0.36	4.35 ± 0.35	4.56 ± 0.45	4.45 ± 0.43	0.025
	WBC (10^3/μL)	5.67 ± 1.15	5.59 ± 1.28	5.33 ± 1.29	5.37 ± 1.53	0.518 [(2)]
	Hemoglobin (g/dL)	13.81 ± 1.07	13.47 ± 1.01	13.67 ± 1.06	13.59 ± 1.05	0.033 [(2)]
	Hematocrit (%)	42.59 ± 3.30	40.63 ± 3.38	42.43 ± 3.67	41.27 ± 3.65	0.072
	Platelet (10^3/μL)	252.18 ± 46.96	230.48 ± 45.72	225.58 ± 42.38	206.32 ± 43.90	0.640 [(3)]
	Neutrophil (%)	49.61 ± 6.91	49.81 ± 8.50	50.90 ± 7.50	50.67 ± 8.02	0.448
	Lymphocyte (%)	39.46 ± 6.19	39.96 ± 8.00	38.48 ± 7.66	38.26 ± 7.96	0.967
	Monocyte (%)	6.80 ± 1.39	6.76 ± 0.98	7.00 ± 1.62	7.28 ± 1.95	0.377
	Eosinophil (%)	2.87 ± 2.26	2.24 ± 1.33	2.25 ± 1.28	2.55 ± 1.72	0.033
	Basophil (%)	1.26 ± 0.37	1.23 ± 0.40	1.31 ± 0.39	1.24 ± 0.36	0.698 [(2)]
	ESR (mmhr)	7.58 ± 7.18	6.98 ± 5.92	7.28 ± 7.84	8.32 ± 8.42	0.195
Biochemical parameters	AST (IU/L)	26.16 ± 6.42	24.64 ± 6.66	24.94 ± 5.99	26.16 ± 7.27	0.008
	ALT (IU/L)	22.34 ± 10.91	19.12 ± 10.43	20.32 ± 6.20	19.64 ± 6.80	0.088
	γ-GTP (IU/L)	19.12 ± 13.21	19.86 ± 12.61	19.82 ± 12.24	20.58 ± 14.84	0.691
	Total protein (g/dL)	7.40 ± 0.40	7.36 ± 0.45	7.26 ± 0.37	7.30 ± 0.34	0.145 [(2)]
	Albumin (g/dL)	4.50 ± 0.23	4.44 ± 0.22	4.40 ± 0.22	4.38 ± 0.23	0.949 [(3)]
	Glucose (mg/dL)	101.00 ± 11.10	96.12 ± 10.13	102.06 ± 11.56	96.56 ± 11.13	0.825 [(2)]
	Total bilirubin (mg/dL)	0.89 ± 0.19	0.88 ± 0.24	0.84 ± 0.30	0.80 ± 0.28	0.465 [(2)]
	Na (mmol/L)	140.44 ± 2.21	141.80 ± 1.84	140.48 ± 2.01	141.68 ± 1.78	0.873
	K (mmol/L)	4.23 ± 0.27	4.28 ± 0.31	4.30 ± 0.34	4.33 ± 0.28	0.787 [(2)]
	Cl (mmol/L)	103.18 ± 1.88	104.16 ± 1.77	103.80 ± 1.63	104.26 ± 1.68	0.232
	Creatinine (mg/dL)	0.74 ± 0.14	0.75 ± 0.12	0.76 ± 0.16	0.75 ± 0.14	0.054
	BUN (mg/dL)	14.96 ± 4.26	15.14 ± 3.92	15.51 ± 3.54	15.27 ± 3.24	0.571 [(2)]
	Uric acid (mg/dL)	4.67 ± 1.14	4.82 ± 1.16	4.78 ± 1.11	4.75 ± 1.25	0.358
	hs-CRP (mg/L)	0.84 ± 0.78	0.71 ± 0.64	0.63 ± 0.53	0.80 ± 1.02	0.276
	TSH (μIU/mL)	2.35 ± 1.20	—	2.40 ± 1.13	—	—

Values are presented as mean ± SD; The *p*-values represent between-group comparisons of changes from baseline to week 12; [(1)] Analyzed by Wilcoxon rank-sum test between the groups; [(2)] Analyzed by independent t-test between the groups; [(3)] Analyzed by ANCOVA (adjusted for baseline values). TSH was assessed at screening only as part of the eligibility assessment. RBC, red blood cell; WBC, white blood cell; ESR, erythrocyte sedimentation rate; AST, aspartate aminotransferase; ALT, alanine aminotransferase; γ-GTP, gamma-glutamyl transpeptidase; BUN, blood urea nitrogen; hs-CRP, high-sensitivity C-reactive protein; TSH, thyroid-stimulating hormone; ANCOVA, analysis of covariance.

Table S2. Changes in vital signs during the 12-week intervention.

**Variables**	**CS Group (*n*=50)**			**Placebo Group (*n*=50)**			***p*-value [1]**
	**Baseline**	**Week 6**	**Week 12**	**Baseline**	**Week 6**	**Week 12**	
Systolic blood pressure (mmHg)	129.80 ± 6.87	126.78 ± 7.24	125.86 ± 8.18	125.32 ± 8.92	125.20 ± 7.95	126.30 ± 7.34	0.320 [2]
Diastolic blood pressure (mmHg)	76.64 ± 7.25	72.12 ± 7.25	72.76 ± 6.68	74.78 ± 9.29	74.22 ± 8.30	73.88 ± 8.82	0.198
Pulse rate (beats/min)	73.08 ± 9.82	77.54 ± 11.47	76.10 ± 10.49	75.80 ± 11.35	73.30 ± 9.38	75.92 ± 11.22	0.272
Body temperature (°C)	36.46 ± 0.08	36.48 ± 0.10	36.51 ± 0.08	36.46 ± 0.08	36.49 ± 0.10	36.51 ± 0.08	0.865 [3]

Values are presented as mean ± SD; The *p*-values represent between-group comparisons of changes from baseline to week 12; [1] Analyzed by independent t-test between the groups; [2] Analyzed by ANCOVA (adjusted for baseline values); [3] Analyzed by Wilcoxon rank-sum test between the groups. ANCOVA, analysis of covariance.
